# Protein Structure Analysis and Prediction with Statistical Scoring Functions

**DOI:** 10.3390/ijms22168665

**Published:** 2021-08-12

**Authors:** Peter Lackner, Markus Wiederstein

**Affiliations:** Department of Biosciences, University of Salzburg, 5020 Salzburg, Austria; markus.wiederstein@sbg.ac.at

The PDB database provides more than 150,000 entries for biological macromolecular structures. The vast majority of the entries comprise proteins. Thus, we can resort to a large dataset that encodes information about sequence–structure–function relationships. Many bioinformatics approaches take advantage of this information and utilize it for a wealth of basic biological, biochemical, and biophysical problems. A well-established key approach is the statistical analysis of experimentally resolved structures for the subsequent derivation of statistical scoring functions (SSFs, also referred to as statistical energy functions, knowledge-based potentials, or mean force potentials). Such SSFs are employed in numerous bioinformatics methods, e.g., for the assessment of experimentally determined structures or in the prediction of 3D protein structures, protein–protein interactions, protein–ligand interactions, protein stability, etc.

The original purpose of what is now termed “statistical scoring function” was the description of molecular interactions. Different authors established energy-like functions depending on the distance between two atoms, dihedral angles, density of neighboring atoms (contacts), or orientation and distance. Meanwhile, SSFs are viewed in more general ways by certain authors, which is also reflected in distinct contributions to this Special Issue.

Georgoulia and Bjelic investigated the prediction of protein–protein binding interactions in dimeric coiled coils. For this purpose, they applied Rosetta, a versatile set of computational methods for molecular modeling and analysis developed at David Baker’s lab. The heart of Rosetta are molecular scoring functions that combine physics-based terms such as Van der Waals interactions with statistics-based terms such as a distance-dependent atom pair potential.

Laimer and Lackner contributed a method named MHCII3D that concentrates on the structure-based prediction of peptides that bind to MHC II molecules. Biologically, such an interaction is part of natural antigen recognition and triggers immune responses. This process plays also a key role in diseases such as allergies and cancer. MHCII3D employs comparative modeling and a distance-dependent SSF for the prediction of potential binding peptides. In contrast to competitor methods, it does not employ machine learning and is therefore not constrained by learning data from wet lab binding experiments.

Poot Velez et al. describe a new way of representing protein structure and apply it to the prediction of protein–protein interactions (PPIs). They map the atomic three-dimensional structures of proteins participating in protein–protein interactions to compact representations based on 26 residue cluster classes (RCCs). Variants of combining the individual RCCs of binding partners are then analyzed with respect to their ability to correctly classify PPIs with machine learning methods.

Alshehri et al. present the first large-scale characterization of intrinsically disordered proteins in the proteome of the genus Camelus. Here, SSFs are used (in addition to other methods) to predict disordered proteins/protein regions in Camelus species and Homo sapiens. Those proteins are then functionally annotated and systematically compared with respect to their degree of disorder and their biological roles. The authors highlight several functional categories that are overrepresented among Camelus disordered proteins when compared to the human proteome.

The applicability of SSFs derived from known protein structures is naturally challenged by so-called xenoproteins, which are built from amino acids not part of life’s standard alphabet. Mayer-Bacon et al. review empirical work on the incorporation of non-canonical amino acids into proteins and juxtapose these findings with more theoretical considerations about the properties of amino acids from an evolutionary perspective. The review stimulates rethinking of many of the assumptions underlying current prediction models.

